# REM sleep respiratory behaviours match mental content in narcoleptic lucid dreamers

**DOI:** 10.1038/s41598-018-21067-9

**Published:** 2018-02-08

**Authors:** Delphine Oudiette, Pauline Dodet, Nahema Ledard, Emilie Artru, Inès Rachidi, Thomas Similowski, Isabelle Arnulf

**Affiliations:** 1 Sorbonne Université, IHU@ICM, INSERM, CNRS UMR7225, équipe MOV’IT, F-75013 Paris, France; 2 AP-HP, Hôpital Pitié-Salpêtrière, Service des Pathologies du Sommeil (Département “R3S”), F-75013 Paris, France; 3 Sorbonne Université, INSERM, UMRS1158 Neurophysiologie Respiratoire Expérimentale et Clinique, F-75013 Paris, France; 4 AP-HP, Hôpital Pitié-Salpêtrière, Service de Pneumologie et Réanimation Médicale (Département “R3S”), F-75013 Paris, France

## Abstract

Breathing is irregular during rapid eye-movement (REM) sleep, whereas it is stable during non-REM sleep. Why this is so remains a mystery. We propose that irregular breathing has a cortical origin and reflects the mental content of dreams, which often accompany REM sleep. We tested 21 patients with narcolepsy who had the exceptional ability to lucid dream in REM sleep, a condition in which one is conscious of dreaming during the dream and can signal lucidity with an ocular code. Sleep and respiration were monitored during multiple naps. Participants were instructed to modify their dream scenario so that it involved vocalizations or an apnoea, -two behaviours that require a cortical control of ventilation when executed during wakefulness. Most participants (86%) were able to signal lucidity in at least one nap. In 50% of the lucid naps, we found a clear congruence between the dream report (*e*.*g*., diving under water) and the observed respiratory behaviour (*e*.*g*., central apnoea) and, in several cases, a preparatory breath before the respiratory behaviour. This suggests that the cortico-subcortical networks involved in voluntary respiratory movements are preserved during REM sleep and that breathing irregularities during this stage have a cortical/subcortical origin that reflects dream content.

## Introduction

Breathing, although a cyclical phenomenon, appears quite variable when we are awake. In addition to an intrinsic breath-by-breath variability of resting breathing thought to derive from the complex behaviour of the respiratory central pattern generators^1–4^, our breathing is heavily influenced by our activities and emotions. The breathing automatisms can also be disrupted by voluntary actions, either respiratory (*e*.*g*., voluntary apnoeas as in diving or voluntary hyperventilation) or non-respiratory (speaking, playing music on a wind or brass instrument). This is allowed by the characteristically dual neural control of breathing, with subcortical and cortical networks in constant interplay^5–8^.

Contrasting with what occurs during wakefulness, breathing becomes more and more regular while we dive into deeper stages of non-rapid eye movement (NREM) sleep^9^. Breathing is, in contrast, erratic in REM sleep, during which brief central apnoeas may occur^10^. REM sleep ends a sleep cycle and is characterized by cortical activation, muscle paralysis (atonia), and rapid eye movements (characterizing phasic REM sleep); rich mental imagery (dreams) often accompanies this stage^11^. Why is respiration so erratic during REM sleep?

REM sleep irregular breathing cannot be explained by chemoreceptor^12^ or vagal afferent activities^13^ because the ventilatory responses to chemical stimuli and other respiratory reflexes are blunted during periods of REM sleep with eye movements (phasic REM sleep)^14^. In cats, the activity of medullary respiratory neurons increases in REM sleep compared to NREM sleep, suggesting the existence of an excitatory drive to the respiratory central pattern generators in REM sleep^15^. This excitatory drive persists when spontaneous breathing is eliminated by mechanical ventilation^16^, but its neural origin is still unclear. Pontine-dependent processes could cause the co-activation of the multiple phasic events that occur during REM sleep. In support of this idea, discharge rates of medullary neurons, whose activity is highly specific to REM sleep, correlate with eye movement bursts and erratic breathing^17^. Furthermore, the activity of medullary respiratory neurons in REM sleep is related to the density of ponto-geniculo-occipital waves, a hallmark of REM sleep^18^. However, pontine cats, in which the structures above the pons have been removed, have regular (and not irregular) breathing in REM sleep; this suggests that supra-pontine structures cause the breathing irregularities observed in normal cats in REM sleep^19^. The nature of these structures is not known.

Many supra-pontine structures modulate the respiratory neural outflow generated by the central respiratory pattern generators during wakefulness. These structures include the caudal hypothalamus, the periaqueductal grey area and midbrain regions^20–22^, all of which are involved in the coupling of respiration with locomotion^21^. Limbic and paralimbic networks comprising the amygdala, the anterior and dorsal cingular cortices, and the insular cortex^23^ are involved in emotional respiratory modulation. Finally, cortico-subcortical networks can also influence breathing control, through bulbar projections^24^ or through direct corticospinal projections bypassing the central pattern generators^25^. In this way, modifications of breathing can be observed in response to mental imagery during wakefulness where, for example, imagining a physical exercise (*e*.*g*., rowing) can suffice to increase ventilation^26–28^.

In keeping with ventilation changes observed during imagined exercise, our general working hypothesis advocates that REM sleep-related breathing irregularities depend on cortical projections to medullary and/or spinal respiratory motoneurons and are correlated with the mental imagery of REM-sleep dreams.

Whether the phasic properties of REM sleep, such as eye movements or respiratory events, are related to dream imagery rather than random markers of brainstem activation has been intensely debated since the discovery of REM sleep^29^. In a previous study, large respiration amplitude, but not respiration variability, correlated with intensive active participation in the dream^30^. In another experiment, participants’ likelihood of reporting a dream after REM sleep was positively correlated with the rate and variability of the respiration immediately preceding the awakening. This relationship was even stronger if the mental content was judged vivid, emotional, and involved physical activity^31^.

Such *a posteriori* correlations have important methodological biases due to their retrospective nature, including forgetting, reconstruction of missing fragments, censorship^32^ and the impossibility of temporally coupling a dream report with its corresponding segment in the sleep recording. Plus, sleepers have usually no control over the dream scenario, so the experimenter can only hope to receive dream reports linked to the question of interest.

To circumvent these difficulties, we used a unique model: lucid dreamers with narcolepsy. Contrary to what occurs in common dreams, sleepers engaged in a lucid dream are aware that they are dreaming and can even exert control over the dream scenario^33,34^. Because eye movements are spared the muscle paralysis characteristic of REM sleep, lucid dreamers can ‘communicate’ with an ocular code, thus allowing the isolation of the beginning and the end of a dream episode on the physiological sleep recording. Lucid dreaming is a challenging state to reach, especially in a laboratory setting^34^. Most published studies show only a limited number of lucid dreams, collected in a few well-trained subjects over multiple nights^35–37^.

Patients with narcolepsy experience excessive daytime sleepiness and manifest abnormal transitions between wakefulness and REM sleep, including rapid entry into REM sleep, cataplexy, hypnagogic hallucinations and sleep paralysis^38^. They are also natural, potent lucid dreamers; our patients were capable of performing multiple lucid dreams during a few daytime naps in our laboratory^39^.

To support the notion that REM sleep respiratory irregularities reflect dream content through cortical inputs, we asked lucid dreamers with narcolepsy to direct their dreams towards a pre-defined scenario involving respiratory-related mental content (typically a central apnoea). We then analysed whether respiratory behaviours collected in real time matched with the dream content.

## Results

Participants were asked to influence their dream content when they reached lucidity. Depending on the nap, they had to imagine a scenario involving an apnoea (*e*.*g*., diving under water), vocalizing or yodelling in their dreams. They had to signal the beginning and the end of the action with the pre-defined ocular code (left-right-left-right). These types of respiratory behaviours involve descending inhibitory cortical inputs to the medullary respiratory central pattern generators^24^ to produce breath holding (in the case of voluntary apnoea) or to avoid the unwanted interruption of a vocal utterance by automatic inspiration (in the case of speech). They can also involve excitatory corticospinal inputs bypassing the medullary respiratory pattern generators^25^ to produce preparatory breaths^8^. In the hypothesis of a congruence between respiratory behaviours during REM sleep and corresponding respiratory mental content, the descending inhibitory inputs should manifest as central apnoeas during sleep and respiration monitoring, and the excitatory inputs as changes in the characteristics of the breath immediately preceding such apnoeas (see methods).

### Lucid REM sleep episodes

The 21 patients rapidly fell asleep and reached REM sleep in most naps, as expected in narcolepsy; 74/98 naps contained REM sleep, whereas the other 24 only included NREM sleep. Patients fell asleep in 3.3  ±  2.8 min, and REM sleep latency was 9.1  ±  8.4 min.

Furthermore, 86% of patients (18/21) clearly succeeded, at least once, in signalling lucidity during REM sleep with the pre-established ocular code. Among the 74 REM sleep naps, 32 included the ocular code at least once. In total, we were able to identify 94 clear ocular codes, with the number of ocular codes per patient across the multiple naps ranging from 1 to 16.

**Figure 1:**
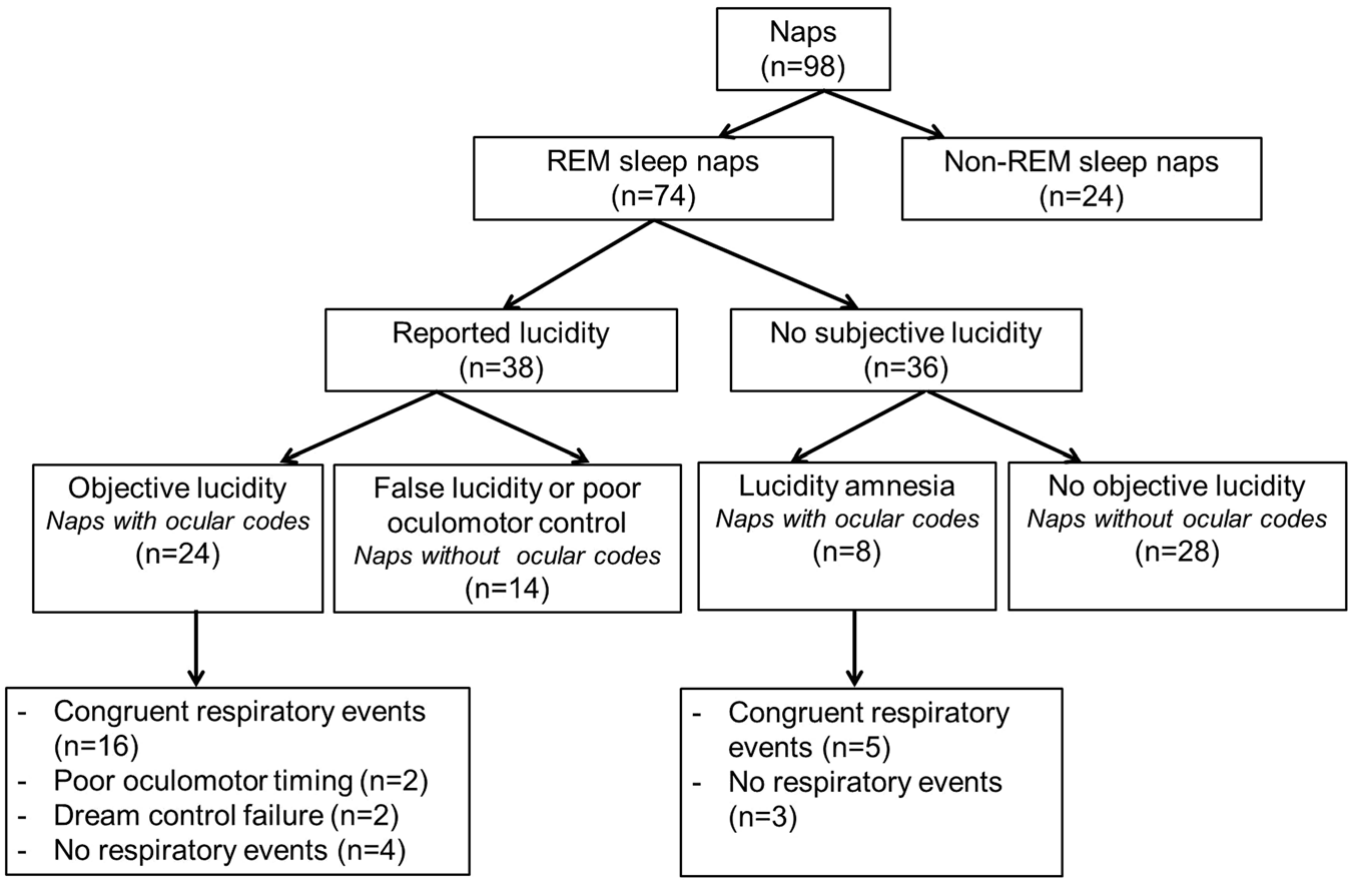
Summary of the different degrees of lucidity mastery achieved across all recorded naps.

During the 74 REM sleep naps, patients with narcolepsy varied in their lucidity level, their abilities to control the dream world (mental content) and the real world (ocular action), as well as the recollection of their sleep experience afterwards (Fig. 1). Together, the results are in line with a relationship between respiration during REM sleep and mental content, but the weight of evidence differs from one nap to another, as summarized in Fig. 1 and described in more detail below.

### Successful task execution in lucid dreams: unambiguous evidence for a congruence between subjective experience and observed physiology

In the 32 coded naps, 50% included an ocular code preceded or followed by a visible respiratory marker (large inspiration or central apnoea) congruent with the dream report collected afterwards, clearly confirming a match between respiration and dream content.

These congruent episodes were distributed over the course of 16 naps (from 11 different patients), with 1 to 3 events/nap, and a total of 22 coded events (ocular code plus respiratory behaviour).

**Figure 2:**
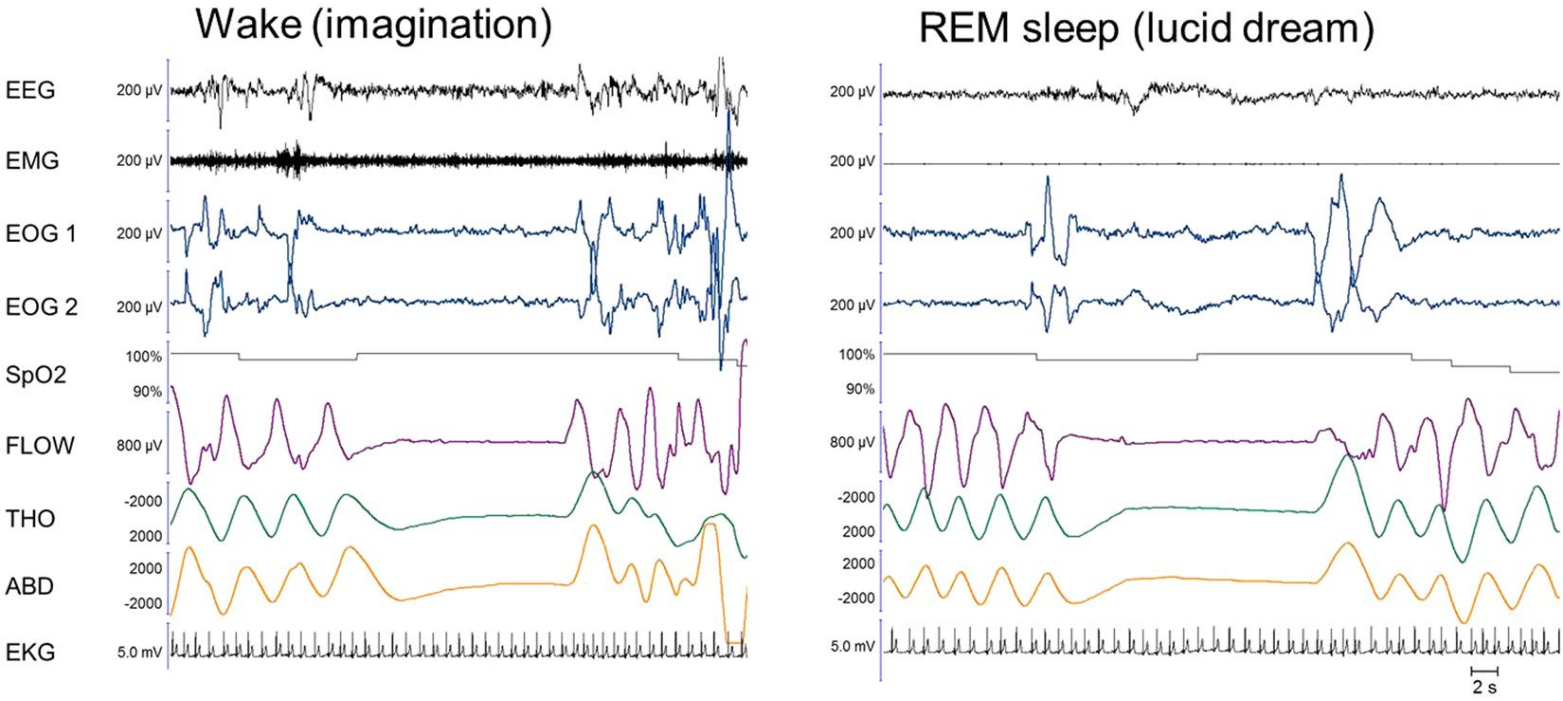
Example of an apnoea executed during an imagined diving task during wakefulness (left panel) and in a lucid dream during coded REM sleep (right panel). In the right panel, the absence of muscle tone in the EMG and the theta frequency on the EEG confirmed that the patient was in REM sleep during the lucid dream. Between the two ocular left-right codes, we observe an 11-second-long central apnoea, characterized by a cessation of nasal flow along with the absence of thoracic and abdominal movements and a mild heart rate decrease. After this episode, the patient reported a dream in which he was threatened by a gun and held his breath in fear. Note that in the imagined condition (left panel), the last breath before the apnoea appears slightly different from the previous ones regarding its time dynamics, but there is no evidence of augmented amplitude. In the lucid dream condition, there is no evidence of pre-apnoeic preparation. EEG: electro-encephalogram; EMG: electromyogram; EOG1 and EOG2: electro-oculogram for left and right movements, respectively; SpO2; transcutaneous oxygen saturation; FLOW: nasal flow; THO: thoracic movements; ABD: abdominal movements; EKG: electro-cardiogram

**Figure 3:**
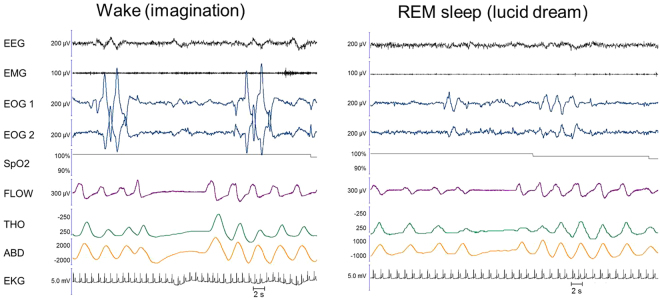
Example of yodelling executed in imagination during wakefulness (left panel) and in a lucid dream during sleep (right panel). A patient, who performed singing exercises along with her sister and a young version of herself in her dream, performed a 9-second-long central apnoea surrounded by two ocular codes in REM sleep (right panel). Note how similar the breathing pattern is in the lucid dream and during a similar behaviour executed in imagination during *wakefulness*. In the imagined condition, there is clear evidence for “pre-phonatory” preparation, with the last breath before the central apnoea pattern showing greater amplitude and sharper dynamics than the previous ones on the flow tracing. This pattern was not present in the lucid dream condition.

**Figure 4:**
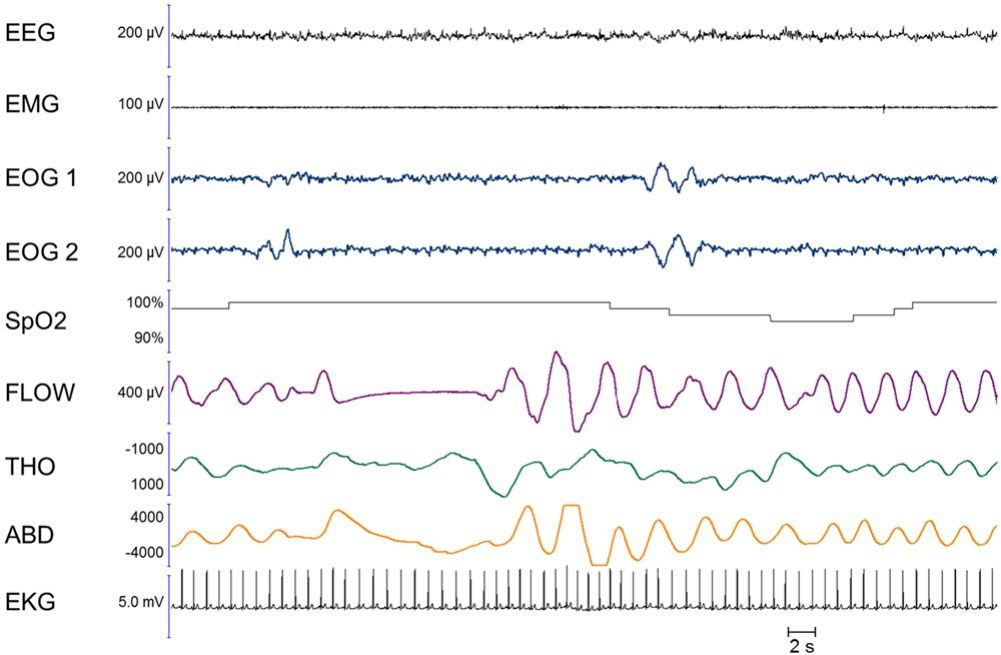
Additional example of an apnoea executed in a lucid dream during sleep. A patient performed a 9-second-long central apnoea surrounded by two ocular codes in REM sleep. This respiratory behaviour was congruent with her subsequent dream report that she held her breath to avoid smelling a poisonous odour while walking in the city’s subway. The breath immediately preceding the apnoea appears bigger and sharper than the previous ones on the flow tracing and the abdominal displacement tracing, suggesting preparation/planning.

**Figure 5:**
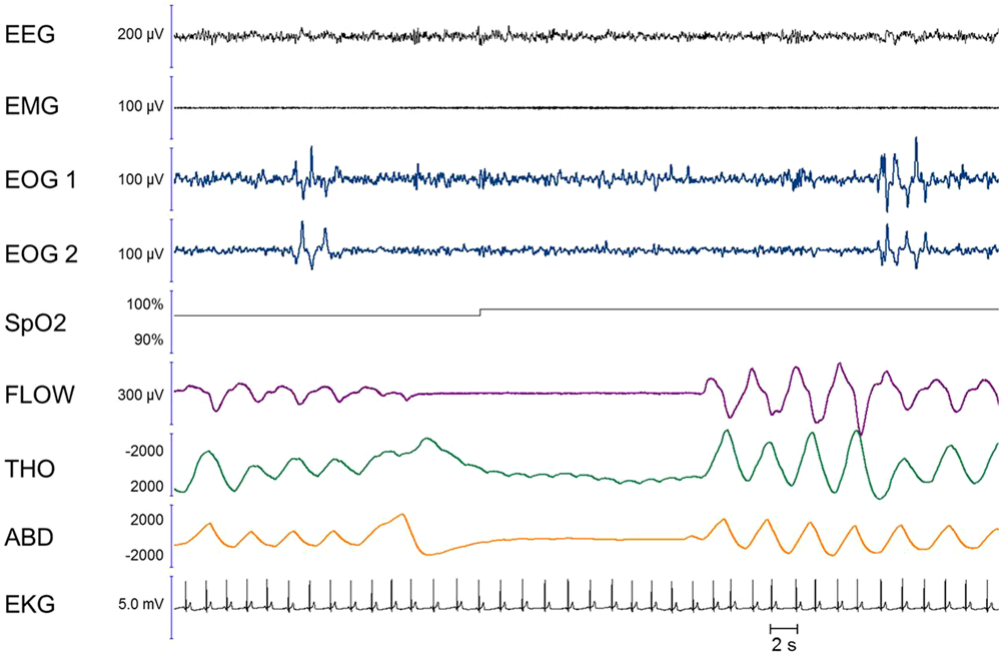
Additional example of an apnoea executed in a lucid dream during sleep. In this REM sleep episode, we observed a 21-second-long central apnoea bordered by two ocular codes. The subject remembered that he performed the task in the dream but could not report what he dreamt about. The breath immediately preceding the apnoea shows a higher amplitude than the previous breaths on the abdominal tracing, suggesting preparation. Note the oscillations related to heart rate on the thoracic trace during the apnoea; these oscillations are not visible on the nasal flow signal, which suggests that the glottis was closed during the event.

Notably, we observed the ocular code surrounding the respiratory dream, both before and after, in 8/16 patients naps (apnoea dreams, n  =  6; vocalisation dreams, n  =  2). Example lucid dreams recorded via sleep and respiration monitoring are provided in Figs 2–5. The apnoeas lasted from a few seconds to 20 s.

In 6 REM sleep episodes (from different patients), a central apnoea was either preceded or followed, but not both, by an ocular code. A missing ocular code at the end of an episode could indicate a loss of lucidity, which progressively vanished as the dream progressed. In contrast, a missing ocular code at the beginning of the episode could be explained by a late recollection of the instructions, *e*.*g*., the subject suddenly remembered that she had to indicate lucidity and performed an ocular code while she was in the middle of an apnoea. An incomplete control of eye movements could also be involved in the missing ocular signals.

In 2 REM sleep episodes, an ocular code preceded a respiratory event, but the event was directly followed by an arousal. In these cases, it is unclear whether it was lucidity itself, the fact of signalling lucidity with voluntary eye movements, or an unrelated event that triggered awakening.

Among the 22 respiratory behaviours accompanied by ocular codes and congruent with the dream report afterwards, 10 were preceded by a visible preparatory breath (see methods), an unequivocal indicator of voluntary breath holding. No clear signs of preparation were visible on the respiratory markers for the 12 remaining events.

### Limits of lucid dreaming ability: ambiguous evidence for a congruence between subjective experience and observed physiology

In the other 14 coded naps, patients failed to control the dream content while lucid (n  =  2), poorly timed their ocular code in regard to the respiratory event (n  =  2), or did not recall that they performed the ocular code upon awakening (n  =  3) or that they performed the entire task (respiratory behaviour plus ocular code, n  =  5); ultimately, no respiratory event was clearly identifiable on the respiratory markers (n  =  4).

#### Dream control failure

Two patients reported that they tried to do the task but failed to do so; one jumped from a cliff but forgot to stop breathing, and another tried to speak but could not produce any sound. This showed that controlling the dream scenario is not an easy task, even in experienced lucid dreamers.

#### Poor oculomotor timing

In 2 additional REM sleep episodes, patients asserted that they both performed the task and properly signalled it. In these cases, ocular codes and central apnoeas were indeed visible in the sleep recording, but there were no signs of respiratory changes in the vicinity of the ocular codes. One patient was (in her dream) a stuntwoman whose job was to extract herself from a burning car. She held her breath while passing through the flames, then held her breath in fear. Patients could have done something else between the ocular code and the dream associated with apnoea (*e*.*g*., walking towards the car location first) or simply forgot to signal lucidity at the right time, two conditions that would explain the temporal mismatch between the two events; alternatively, patients could have performed the task immediately after the ocular code, in which case the dream content and the observed respiratory behaviour did not match.

**Figure 6:**
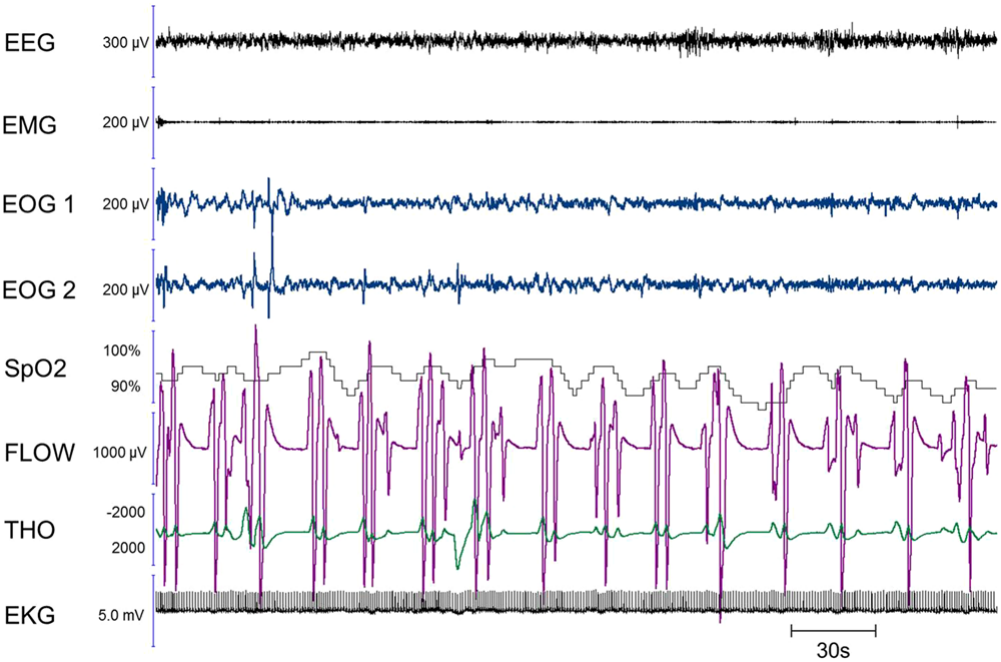
Multiple apnoeas executed during REM sleep, with non-locked ocular codes. A patient who dreamt that she was swimming under water, performing racing breaststrokes (repetitively putting her head in and out of the water), exhibited regularly repeating central apnoeas on the sleep recording, each preceded by ample respirations (hyperventilation). While ocular codes were present in the corresponding nap, they were not temporally locked to the beginning and the end of this multiple apnoea episode. However, this recording provides very spectacular evidence in favour of the link between breathing irregularities during REM sleep and the presence of imaginary actions during the corresponding bout of dreams. The figure shows a 5-min period.

Interestingly, all 3 coded respiratory events (distributed over 2 naps) were preceded by ample breaths (hyperventilation pattern, see Fig. 6), which might be a consequence of imaginary action during dreams, as imaginary actions during wakefulness are accompanied by an increased ventilatory rate^26,40,41^.

#### Sleep experience amnesia

In 5 episodes, we observed central apnoeas, delimited by an ocular code, but the patients had no recollection of having performed the task in their dreams or signalling lucidity. This suggests amnesia for the lucid period. Note that participants were not awakened directly after the episode in this experiment, as they were allowed to sleep for 30 min straight. This additional time between the lucid dream and awakening could explain why the memory for the lucid episode faded in some patients. Interestingly, none of these episodes was preceded by a preparatory breath.

We observed the presence of ocular codes in 3 additional REM sleep naps. After these naps, patients either reported a dream unrelated with the task (*e*.*g*., playing football with a plastic spinning top) or no dream at all. There was no visible respiratory behaviour in the vicinity of the ocular codes in these cases. When questioned on their lucidity during the nap, patients all said they did not perform the ocular code, suggesting that they forgot part or all of what occurred during their sleep.

#### Incongruence between subjective behaviours and objective evidence

There were 4 instances of coded lucid dreams without any visible respiratory adaptation. In particular, 2 patients vocalized in dreams; one called ‘Leo’ and the other screamed. It is possible that the vocalizations were too short to elicit a visibly prolonged expiration on the recording.

### Non-coded REM sleep episodes

After 14 REM sleep episodes, patients asserted that they were lucid, had a respiratory dream and signalled it, but we could not detect any clear ocular codes. For 5/14 of these episodes, participants might actually have performed some ocular codes, but they were incomplete or unclear, so we decided to discard them and considered the nap as ‘non-coded’. Nevertheless, these doubtful events were all accompanied by congruent respiratory measures. One patient said he was running in the mountains then swimming under water and attempted to do the ocular code but was not sure whether he had succeeded doing so; we observed a central apnoea and a mild heart rate acceleration just before an arousal, without any ocular codes. It is possible that these patients indeed achieved lucidity, but that their eye movements were not controlled enough to translate into reality.

For the other 9 REM sleep episodes, we could not observe any ocular code or respiratory change in the sleep recordings, in contradiction with patients’ reports. These findings could indicate that respiratory changes did not parallel dream content in these cases. Alternatively, patients could have reached lucidity in NREM sleep rather than REM sleep, and thus have been unable to signal lucidity with the ocular code.

Finally, in the last 28 REM sleep episodes, patients had no memory of having dreamt or performed the ocular code. In agreement with their subjective report, we could not find any ocular signal or central apnoea during these naps.

## Discussion

In this study, we hypothesized that respiratory irregularities observed during REM sleep were driven by cortical or subcortical inputs and reflected mental content. Our finding that most patients were able to influence their respiratory behaviour during lucid REM sleep supports this hypothesis, at least for the particular case of central apnoeas. Indeed, our lucid sleepers achieved voluntary apnoeas, as confirmed by the ocular codes surrounding them and their long duration (typically between 10 and 20 s), which is unusual for spontaneous central apnoeas in REM sleep. Moreover, some apnoeas shown here started by a minimal increase in nasal flow associated with a thoracic and abdominal collapse, and the heart signal did not propagate to the nasal pressure; these constitute two signs of glottis closure, a mechanism that occurs mostly during voluntary apnoeas (see Fig. 5). When a voluntary apnoea is performed awake, several cortical and subcortical networks are recruited, as shown in fMRI. They involve the basal ganglia, insula, frontal and parietal cortex, and thalamus, in addition to the pons. It has been hypothesized that the pons integrates information from supra-brainstem structures, and in turn exerts an inhibitory effect on medullary respiratory neurons that lead to breathing cessation^24^. The fact that voluntary apnoeas are possible during lucid dreams suggest that this cortico-pontine drive is maintained during REM sleep. In some instances, we also found preparatory breaths before the coded central apnoea in lucid dreams, suggesting that excitatory cortico-spinal inputs necessary for such preparation while awake are also preserved during REM sleep^8^. Notably, this also suggests a planning of the action, suggestive of a functional connectivity between the premotor and the motor cortex^42^. This is the case for prepared pre-phonatory breaths during awake speech, which are associated with electroencephalographic signs of activation of the supplementary motor area^8^. It has been shown that intracortical connectivity can be preserved (or restored with reference to NREM sleep) during REM sleep^43^. Our observation of preparatory breaths before the coded apnoea in lucid dreams supports this notion.

Do breathing irregularities during REM sleep reflect mental content? Confirming with a much larger sample some anecdotal reports^44–46^, we found a clear congruence between respiratory behaviours observed during lucid REM sleep and the corresponding respiratory mental content in 50% of the lucid naps (16/32), the behaviours in the remaining naps being harder to interpret due to the difficulties inherent in the lucid dreaming model (see below for discussion). Thus, it is tantalizing to conclude that breathing irregularities during REM sleep indeed reflect the richness of dream imagery.

However, the instructions clearly hinted at the goal of the study. It is thus possible that a central apnoea would not have occurred in a spontaneous ‘respiratory’ lucid dream in the absence of instruction. In support of this limitation, some participants reported that they ‘forced’ themselves to do an apnoea while it was not necessary for the dream course (*e*.*g*., a few participants told us they could breathe under water when swimming in their dreams but chose to hold their breath for the experiment). By contrast, there were several instances in which the apnoea was well integrated in the scenario without the participant explicitly recalling the instruction at the moment the apnoea occurred (*e*.*g*., holding one’s breath to avoid smelling a foul odour). In sum, breathing irregularities during REM sleep in our study could be the consequence of the following: (i) voluntary apnoeas, unrelated to mental content; in this case, the mere fact that voluntary apnoeas are possible during REM sleep is still informative as it strongly suggests that some of the structures and circuits generating respiratory movements during REM sleep in general are located rostral to brainstem structures; or (ii) voluntary apnoeas and other respiratory consequences of dreams’ imaginary actions. These two possibilities are difficult to disentangle in the context of this study. However, the presence of a coordinated alternation of central apnoeas and bouts of hyperventilation congruent with dream content justifying such a pattern (see Fig. 6) is compatible with the second possibility, if we extrapolate from what occurs during wakefulness imagination. Indeed, imaginary motor actions are known to activate a large cortical and subcortical network^47^ and are accompanied by an increase in ventilatory rate^26,40,41^.

Beyond the exploration of the structures and circuits modulating breathing during REM sleep, this study also offers a proof of concept that lucid dreamers with narcolepsy provide an interesting model in which to study physiological and cognitive aspects of REM sleep. Here, 86% of lucid dreamers with narcolepsy were able to achieve lucidity in at least one nap during a single day of testing in the lab. When combining all the naps containing REM sleep, 43% included the ocular code signalling lucidity. This score is particularly high when considering how difficult it is to record lucid REM sleep in healthy subjects. For example, among 6 healthy lucid dreamers, who had been trained in lucid dreaming for several years, only one subject was able to perform the requested task and the ocular code after 3 full nights in an fMRI scanner^36^.

Despite our high number of lucid naps, 50% were hard to interpret. This is because the lucid dreaming model still presents inherent difficulties; patients not only had to fall asleep and reach lucidity, they also had to modify the dream scenario, control their eye movements in such a way that the ocular code was produced at the right time (before and after the requested dream) and observable on the electro-oculographic recording (so that the eyes in dreams moved along with the eyes in reality), and, finally, they had to remember and report this lucid episode with enough details for the experimenter to match the dream with physiological measures. During our experiment, we observed that lucid dreamers could fail at any of these steps, preventing firm conclusions for these ambiguous episodes. This suggests that lucid dreaming is not a uniform state but rather a continuum along which different levels of ‘mastery’ can be reached.

Our study had some limitations. First, one could argue that lucid dreaming is a particular case that cannot be generalized to common dreams and to non-lucid REM sleep. We have already reported that some markers of REM sleep, such as arousal index, REM sleep duration, and muscle atonia, remain unchanged between lucid and non-lucid REM sleep^39^. Furthermore, H reflexes, which are caused by an inhibition of the spinal lower motor neurons, are higher during lucid than non-lucid REM sleep^48^, suggesting that the lucid dreaming stage is closer to a deep form of REM sleep than to wakefulness. Second, whether our findings are restricted to lucid REM sleep in narcolepsy could not be evaluated in the absence of a control group without a sleep disorder. However, our sleep recordings are very similar to those observed in previous studies of healthy subjects^46^. We think that narcolepsy is more of a gateway to lucid dreaming, because of the numerous abnormal transitions between wakefulness and REM sleep that characterize this disorder, rather than a particular case of lucid dreaming. Indeed, direct transitions from wakefulness to REM sleep could favour the maintenance of wake metacognition in REM sleep dreams; furthermore, narcolepsy is associated with frightening nightmares and hallucinations that patients might learn early on to control in order to make them less disturbing. Third, evidence for the brain structures involved in breathing irregularities during REM sleep is indirect here, and imaging of the brain structures and connections of interest will be necessary to confirm our conclusions.

All in all, our results speak to a cortical or subcortical origin of breathing irregularities during REM sleep, which possibly reflect underlying mental content.

## Methods

### Participants

Twenty-one patients with narcolepsy recruited among our patients at the sleep disorder clinic took part in the study (11 women; mean age: 31.6  ±  12.4 y.o., range: 17–67 y.o.). One patient came twice. Table 1 indicates demographic and clinical characteristics. All patients met the international criteria for narcolepsy^49^ including the following: (i) excessive daytime sleepiness occurring daily for more than 3 months; (ii) a mean sleep latency shorter than 8 min on the Multiple Sleep Latency Test (5 tests performed at 08:00, 10:00, 12:00, 14:00, and 16:00 after attended night-time polysomnography; (iii) two or more sleep onsets in REM sleep periods during these tests; and (iv) no other causes for these findings. Most participants (76%) had cataplexy (narcolepsy type 1), as identified by clinical interview, and in rare cases, there was direct observation of sudden loss of axial muscle tone with transient absence of deep tendon reflexes. All patients were selected for their exceptional ability to frequently perform lucid dreaming, as mentioned during a clinical interview. The patients treated with stimulants and anticataplectic drugs stopped taking their drugs for 24 h before and during the test.

**Table 1:** Demographic and clinical characteristics of the 21 subjects with narcolepsy.

Variables (Mean ± SD)	N = 21
Age (years)	32.9 [23.4, 38.0]
Gender (% women)	52
Body mass index (kg/m²)	28.3 [22.9, 33.9]
Cataplexy (%)	76
Sleep paralysis (%)	48
Sleep-related hallucinations (%)	67
HLA DQB1*0602 positive allele (%)	76
Disease course (years)	12 [5.8, 26]
Epworth Sleepiness Score at inclusion(0–24)	17 [15, 20]
Multiple Sleep Latency Test at diagnosis:	
Mean sleep latency (min)	3.5 ± 2.4
Sleep onset in REM Periods (n/5)	3.8 ± 2.0

Data presented are median [first quartile, third quartile], mean  ±  standard deviation, or percentage.

The study was conducted according to the French law, with clearance from the local ethics committee (CPP Ile de France VI, # 16–13). The subjects were informed of the purpose of the study and gave written consent.

### Procedure and sleep monitoring of lucid dreaming

Because keeping a dream journal increases both the frequency of dream recall and the chance of accessing lucidity in dreams^50^, patients were asked to keep a dream journal for two weeks prior to the time of the experiment. Subjects were studied in the sleep laboratory during daytime and monitored with video-polysomnography for the entirety of the experiment. The video-polysomnography included 6 EEG channels (Fp1-A2; C3-A2; C3-O1; Fp2-A1; C4-A1; C4-O2), 4 electro-oculography (EOG) channels, 3 surface electromyography channels (*levator menti* and left and right *tibialis anterior* muscles), EKG, pulse oximeter including transcutaneous oxygen saturation measure and pulse wave, thoracic and abdominal belts, nasal pressure sensor, and infrared audio-video recordings. The four EOG electrodes were located along the horizontal (adjacent to the lateral canthi) and vertical planes (left supra- and infraorbital ridges), intersecting the pupil in straight-ahead gaze. EOG1 was the horizontal left electrode, EOG2 the horizontal right one, EOG3 was the left vertical infraorbital electrode, and EOG4 the vertical supraorbital one. EEG signals were acquired in monopolar mode following a 200 Hz sampling. All signals were acquired simultaneously on Brainet software (Médatec Ltd, France).

Depending on their sleepiness level, subjects performed 3 to 7 naps of 30 minutes each. Before each nap, subjects were asked to perform one task involving the voluntary control of respiration: to hold their breath (apnoea) or vocalize. They had to execute these tasks in three conditions: ‘for real’ during wakefulness, in their imagination, and in a lucid dream during the nap. Notably, for the imagination and dream conditions, the apnoea task had to be fully integrated in a coherent dream (*e*.*g*., diving under water) rather than just executed without any connection to the mental content. Participants were instructed to signal their lucidity during REM sleep with an ocular code consisting of a sequence of large horizontal eye movements (left-right-left-right). They had to execute the code before and after doing the task in a dream. At the end of the nap, subjects were asked about their dreams, whether they had been lucid, and if they remembered succeeding at performing the task and making the ocular signal. We did not give them feedback about whether they succeeded at performing the task.

### Polysomnography analysis

Central EEG (C3-A2), EMG and EOGs were used to score sleep stages. Sleep stages, arousals and respiratory events were visually scored in 30-second epochs according to international criteria. Ocular signals were visually targeted, their pattern being significantly different from the REMs of REM sleep (eye movements from the ocular code are of larger amplitude and form a sequence that is unlikely to occur in spontaneous REMs). Doubtful EOG signals were discarded. Respiratory events in REM sleep were visually screened. Two indications of a cortical contribution to breathing control were looked for. They consisted of the following: (1) central apnoeas, defined as a cessation of nasal flow for more than 10 s in the absence of cyclic thoracic and abdominal movements; this pattern is characteristic of a voluntary breath hold; and (2) evidence for the preparation of central apnoeas (“preparatory breaths”), defined as an increase in the amplitude or a change in the dynamics or morphology of the breath immediately preceding a central apnoea; this pattern is characteristic of certain types of vocalizations, specifically, prepared pre-phonatory breaths (see^8^), or of voluntary “protective apnoeas” (namely, those planned to avoid exposure to irritants or noxious smells).
