# Author Correction: REM sleep respiratory behaviours match mental content in narcoleptic lucid dreamers

**DOI:** 10.1038/s41598-018-24179-4

**Published:** 2018-04-12

**Authors:** Delphine Oudiette, Pauline Dodet, Nahema Ledard, Emilie Artru, Inès Rachidi, Thomas Similowski, Isabelle Arnulf

**Affiliations:** 1 Sorbonne Université, IHU@ICM, INSERM, CNRS UMR7225, équipe MOV’IT, F-75013 Paris, France; 2 AP-HP, Hôpital Pitié-Salpêtrière, Service des Pathologies du Sommeil (Département “R3S”), F-75013 Paris, France; 3 Sorbonne Université, INSERM, UMRS1158 Neurophysiologie Respiratoire Expérimentale et Clinique, F-75013 Paris, France; 4 AP-HP, Hôpital Pitié-Salpêtrière, Service de Pneumologie et Réanimation Médicale (Département “R3S”), F-75013 Paris, France

Correction to: *Scientific Reports* 10.1038/s41598-018-21067-9, published online 08 February 2018

The original version of this Article contained an error in the title, where,

“REM sleep respiratory behaviours mental content in narcoleptic lucid dreamers”.

now reads:

“REM sleep respiratory behaviours match mental content in narcoleptic lucid dreamers”.

In addition, the Discussion section contained an error, where,

“For example, among 6 healthy lucid dreamers, who had been trained in lucid dreaming for several years, only one subject was able to perform the requested task and the ocular code after 3 full nights in a fMRI^36^”

now reads:

“For example, among 6 healthy lucid dreamers, who had been trained in lucid dreaming for several years, only one subject was able to perform the requested task and the ocular code after 3 full nights in an fMRI scanner^36^.”

These errors have now been corrected in the HTML and PDF versions of this Article.

